# Diannexin Protects against Renal Ischemia Reperfusion Injury and Targets Phosphatidylserines in Ischemic Tissue

**DOI:** 10.1371/journal.pone.0024276

**Published:** 2011-08-30

**Authors:** Kimberley E. Wever, Frank A. D. T. G. Wagener, Cathelijne Frielink, Otto C. Boerman, Gert J. Scheffer, Anthony Allison, Rosalinde Masereeuw, Gerard A. Rongen

**Affiliations:** 1 Department of Pharmacology and Toxicology, Radboud University Nijmegen Medical Centre, Nijmegen Centre for Molecular Life Sciences and Nijmegen Centre for Evidence Based Practice, Nijmegen, The Netherlands; 2 Department of Orthodontics and Oral Biology, Radboud University Nijmegen Medical Centre, Nijmegen Centre for Molecular Life Sciences and Nijmegen Centre for Evidence Based Practice, Nijmegen, The Netherlands; 3 Department of Nuclear Medicine, Radboud University Nijmegen Medical Centre, Nijmegen Centre for Molecular Life Sciences and Nijmegen Centre for Evidence Based Practice, Nijmegen, The Netherlands; 4 Department of Anaesthesiology, Radboud University Nijmegen Medical Centre, Nijmegen Centre for Molecular Life Sciences and Nijmegen Centre for Evidence Based Practice, Nijmegen, The Netherlands; 5 Department of General Internal Medicine, Radboud University Nijmegen Medical Centre, Nijmegen Centre for Molecular Life Sciences and Nijmegen Centre for Evidence Based Practice, Nijmegen, The Netherlands; 6 Alavita Pharmaceuticals, Inc., Mountain View, California, United States of America; Cardiovascular Research Institute Maastricht, Maastricht University, The Netherlands

## Abstract

Renal ischemia/reperfusion injury (IRI) frequently complicates shock, renal transplantation and cardiac and aortic surgery, and has prognostic significance. The translocation of phosphatidylserines to cell surfaces is an important pro-inflammatory signal for cell-stress after IRI. We hypothesized that shielding of exposed phosphatidylserines by the annexin A5 (ANXA5) homodimer Diannexin protects against renal IRI. Protective effects of Diannexin on the kidney were studied in a mouse model of mild renal IRI. Diannexin treatment before renal IRI decreased proximal tubule damage and leukocyte influx, decreased transcription and expression of renal injury markers Neutrophil Gelatinase Associated Lipocalin and Kidney Injury Molecule-1 and improved renal function. A mouse model of ischemic hind limb exercise was used to assess Diannexin biodistribution and targeting. When comparing its biodistribution and elimination to ANXA5, Diannexin was found to have a distinct distribution pattern and longer blood half-life. Diannexin targeted specifically to the ischemic muscle and its affinity exceeded that of ANXA5. Targeting of both proteins was inhibited by pre-treatment with unlabeled ANXA5, suggesting that Diannexin targets specifically to ischemic tissues via phosphatidylserine-binding. This study emphasizes the importance of phosphatidylserine translocation in the pathophysiology of IRI. We show for the first time that Diannexin protects against renal IRI, making it a promising therapeutic tool to prevent IRI in a clinical setting. Our results indicate that Diannexin is a potential new imaging agent for the study of phosphatidylserine-exposing organs *in vivo*.

## Introduction

Ischemia/reperfusion injury (IRI) is a major cause of cardiovascular morbidity and mortality [1]. Most sensitive to IRI are organs with a high energy demand and an intricate mircovascular network, such as the kidney. Renal IRI frequently complicates shock, cardiac and aortic surgery, delays graft function after transplantation, and has prognostic significance [2], [3]. Furthermore, renal IRI is a major cause of acute kidney injury (AKI), and is commonly observed in *e.g.* renal artery stenosis, sepsis, and various types of renal surgery [4], [5]. Even though IRI is an important and common clinical problem, effective strategies to reduce this condition are inadequate and novel therapies are needed.

The characteristic pathologic changes associated with IRI induced AKI are classically believed to be acute tubular necrosis and interstitial inflammatory cell infiltration [6], [7]. In addition, the role of microvascular dysfunction in AKI has generated increasing interest [8]. The persistence of decreased blood flow after reperfusion (the ‘no-reflow’ phenomenon) is thought to play an essential role in AKI development, especially in the corticomedullary junction and outer medulla. The activation of endothelial cells in microcirculatory vessels is an important event in the inflammatory response upon IRI. Particularly, the translocation of phosphatidylserines (PS) to the outer leaflet of the endothelial cell plasma membrane appears to play a central role in the cascades leading to end-organ damage [9], [10].

Exposed on the endothelial cell surface, PS serve as a binding site for leukocytes, which are able to specifically recognize this phospholipid through a putative PS-receptor or via bridging molecules [11], [12]. Furthermore, PS exposition contributes to pro-thrombinase complex formation and is an important trigger of the complement cascade [13], [14]. As a result, platelets and leukocytes will bind to the activated endothelium and cause an extended pro-thrombotic and pro-inflammatory response, consisting of T-cell recruitment and infiltration of monocytes and macrophages [8], [15]. Overall, adhesion of immune cells will reduce blood flow in the renal microcirculation and cause capillary plugging, thereby aggravating the ischemic injury to renal tubule cells [7].

In light of the detrimental effects of PS exposition, we hypothesize that shielding of PS by PS-binding proteins will inhibit their pro-inflammatory effects, thereby reducing IRI. The endogenous protein annexin A5 (ANXA5) binds with nanomolar affinity to PS in a calcium-dependent manner [16], [17]. ANXA5 forms a 2D crystal structure on PS exposing cell membranes *in vitro*, thereby shielding PS from the extra-cellular environment [18]. For endothelial cells, shielding of PS by ANXA5 was shown to have both anti-thrombotic effects [19], and to prevent leukocytes adhesion [20], making this protein a promising candidate to reduce microvascular dysfunction after IRI. However, rapid renal clearance of ANXA5 limits its clinical utility. In contrast, the synthetic ANXA5 homodimer Diannexin (DA5) was recently claimed to have an increased half-life in the circulation *in vivo* as well as high affinity for exposed PS *in vitro* [21].

The aim of the present study was to investigate the protective role of DA5 in the kidney. We demonstrate for the first time that DA5 ameliorates renal function after IRI, reduces leukocyte influx and tubular damage and reduces renal injury marker expression. Secondly, we determined the short-term biodistribution of DA5 and show for the first time that DA5 targets specifically to tissues undergoing I/R, via high-affinity binding to exposed PS. We conclude that DA5 is a promising candidate to prevent renal IRI, and that these effects are likely mediated through PS-shielding.

## Results

### DA5 reduces tubule damage and leukocyte influx after renal IRI and improves renal function

We investigated the effects of DA5 pretreatment on ischemic kidney damage in a mouse model of bilateral renal IRI. We applied a mild IRI stimulus by inducing 20 min of renal ischemia and either 3 or 8 days of reperfusion. Subsequently, we assessed renal damage, local inflammation and kidney function in mice treated with DA5 or vehicle in comparison to sham-operated mice.

**Figure 1:**
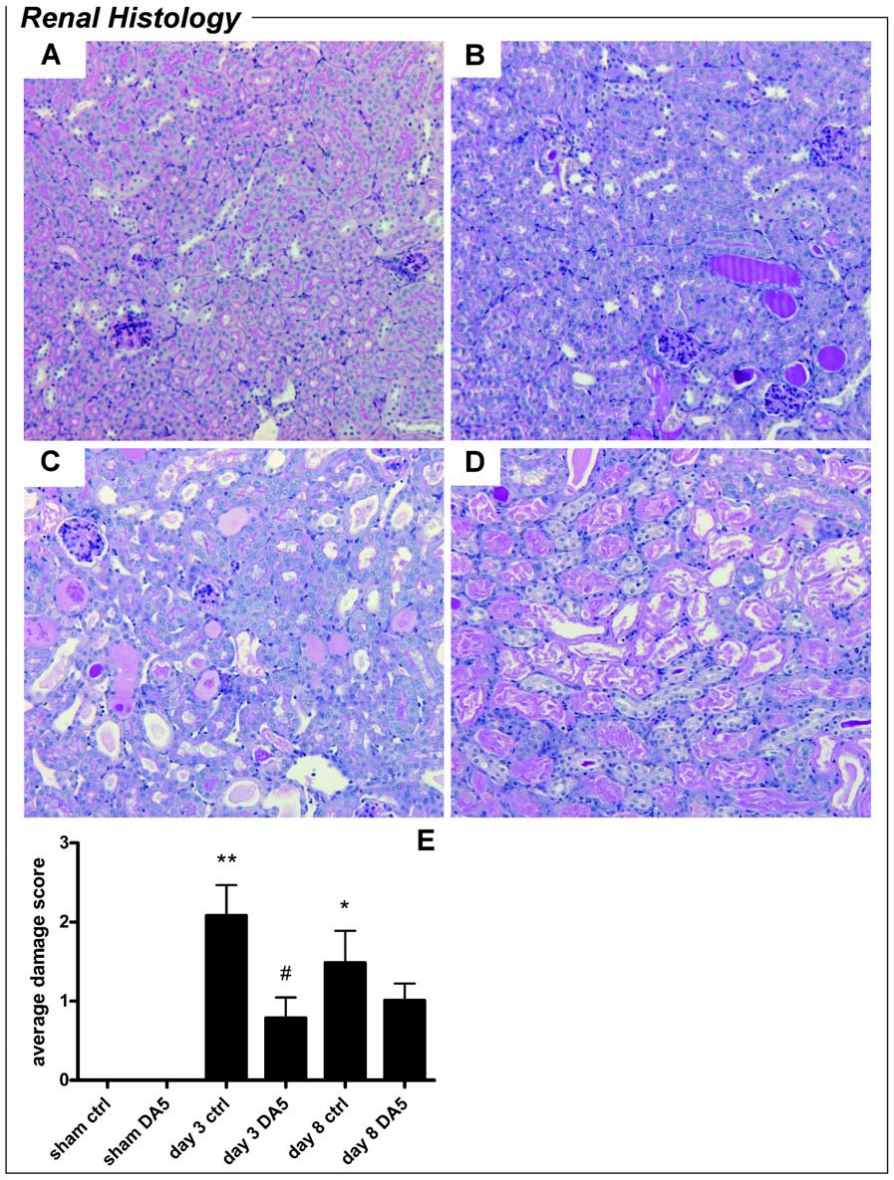
Diannexin (DA5) reduces proximal tubule damage after ischemia-reperfusion injury. (A–D) Representative images of PAS-stained kidney sections (magnification 100x), showing 4 levels of proximal tubule damage after I/R. (A) Damage score 0: intact brush borders and absence of casts or destroyed tubules. Damage score 1 (image not shown) was given for scattered cast formation in less than 1% of tubules. (B) Damage score 2: often clustered cast formation in 5–25% of the tubules. (C) Cast formation and destroyed tubules affecting 40–60% of tubules. (D) Damage score 4: Extensive destruction and cast formation in 70–90% of tubules. Damage score 5 (not observed): destruction or cast formation in 100% of tubules. (E) When compared to sham-operated mice, renal cortex damage was significantly increased in vehicle-treated groups on day 3 and day 8 *post-*I/R. Damage in the DA5-treated groups did not differ significantly from sham-operated mice on day 3 or day 8 *post-*I/R. On day 3, DA5 treatment significantly reduced renal damage when compared to vehicle-treated mice. Values are means ± SEM. *P<0.01, **P<0.001 *vs.* sham group; ^#^ *P<*0.05 *vs.* ctrl group. n = 5 for sham-operated mice and n = 10 for all other groups.

**Figure 2:**
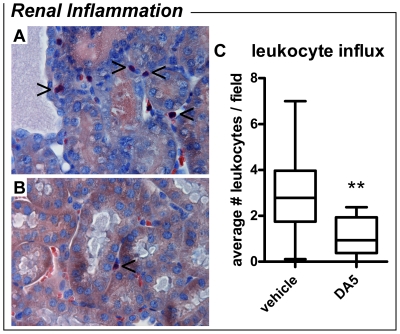
Diannexin (DA5) reduces leukocyte influx 3 days after I/R. Representative pictures of leukocyte influx in (A) vehicle-treated mice and (B) DA5-treated mice (magnification 400x). Granulocytes stained with Leder's esterase are indicated with arrowheads. (C) When compared to vehicle-treated mice, leukocyte influx in renal tissue of DA5-treated mice was decreased on day 3 after IRI induction. Values are means ± SEM. **P<0.01 *vs.* vehicle. n = 8–10 mice per treatment.

Renal proximal tubule damage was quantified by scoring of histological sections (Figure 1). Sham-operated mice lacked any signs of tubular damage and scored 0±0 points. Proximal tubule damage was significantly increased in vehicle-treated mice on day 3 and day 8 *post-*I/R (*P<*0.001 and *P<*0.01, respectively). Damage scores of kidneys from DA5-treated mice did not differ from sham-operated mice on day 3 or day 8. Moreover, DA5 significantly reduced proximal tubule damage on day 3 when compared to vehicle-treated mice (*P<*0.05). Granulocyte influx in the renal cortex was quantified by granulocyte counting in histological sections (Figure 2). On day 3 *post*-I/R, granulocyte influx in the renal cortex of vehicle-treated mice was significantly higher than in DA5-treated mice (*P<*0.01).

**Figure 3:**
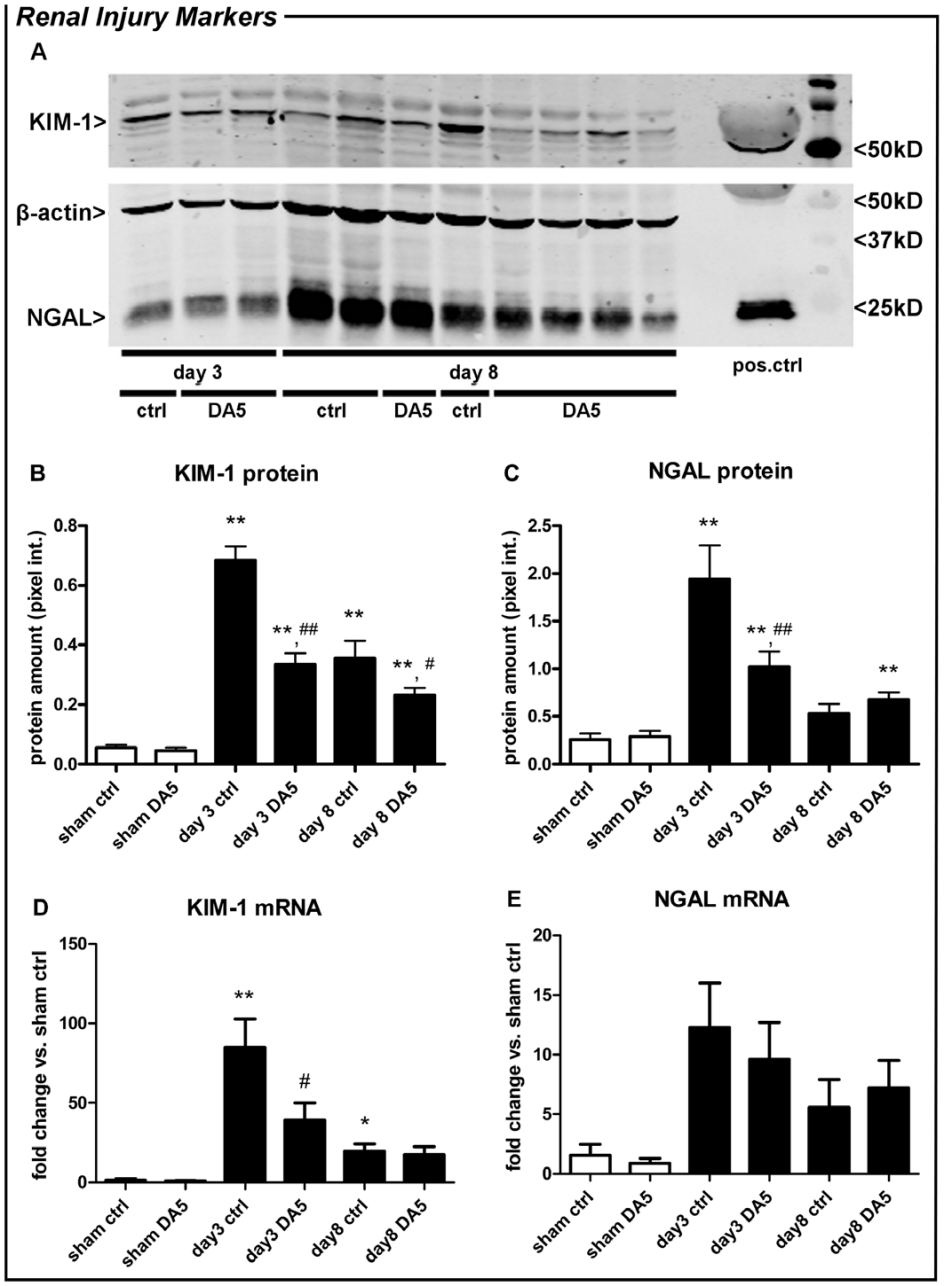
Diannexin (DA5) reduces expression and transcription of renal injury markers after IRI. (**A**) Representative western blot of kidney homogenate from DA5 and vehicle-treated mice (ctrl), after 3 or 7 days of reperfusion, stained for KIM-1, NGAL and β-actin. Purified KIM-1 and NGAL protein were used as positive control. (B,C,D) When compared to sham operation, KIM-1 and NGAL expression, as well as KIM-1 transcription, were increased in renal tissue 3 and 8 days after IRI induction. DA5 treatment significantly reduced protein expression on day 3 (KIM-1 and NGAL) and day 8 (KIM-1), when compared to vehicle-treated mice (ctrl). Also, DA5 reduced the IRI-induced transcription of KIM-1 on day 3 and day 8. (E) NGAL mRNA expression was not significantly elevated on day 3 or day 8 *post*-IRI. CT-values for corresponding β-actin were 17.2±3.2 for KIM-1 and 18.5±2.9 for NGAL. Values are means ± SEM. *P<0.05, **P<0.001 *vs.* sham group; ^#^ *P<*0.05, ^##^ *P<*0.001 *vs.* ctrl group. n = 5 for sham operated mice and n = 10 for all other groups.

We measured protein and mRNA levels of KIM-1 and NGAL, two established biomarkers for renal damage [22], [23]. Western blot analysis showed that KIM-1 and NGAL protein expression was elevated 3 days after I/R in both treatment groups, when compared to sham-operated mice. On day 3, DA5 treatment reduced the I/R-induced KIM-1 and NGAL protein expression by ∼50%, when compared to vehicle-treated mice (*P<*0.001; Figure 3B-C). After 8 days of reperfusion, the increase in KIM-1 protein expression persisted in both treatment groups, but was ∼35% lower in DA5-treated animals (*P<*0.05). NGAL protein expression on day 8 did not differ between treatment groups, but was increased above sham-levels only in DA5-treated mice. In line with the protein levels, KIM-1 mRNA levels were increased in vehicle-treated groups on day 3 and day 8 *post*-ischemia (*P<*0.001 and *P<*0.05, respectively; Figure 3D). In DA5-treated animals, mRNA levels did not differ from sham-operated animals on day 3 or day 8, while KIM-1 mRNA expression on day 3 was significantly lower than in vehicle-treated mice (*P<*0.05). For NGAL, no significant increase in mRNA expression could be detected (Figure 3E), probably because this marker reached its maximum transcription at an earlier time-point [22].

**Figure 4:**
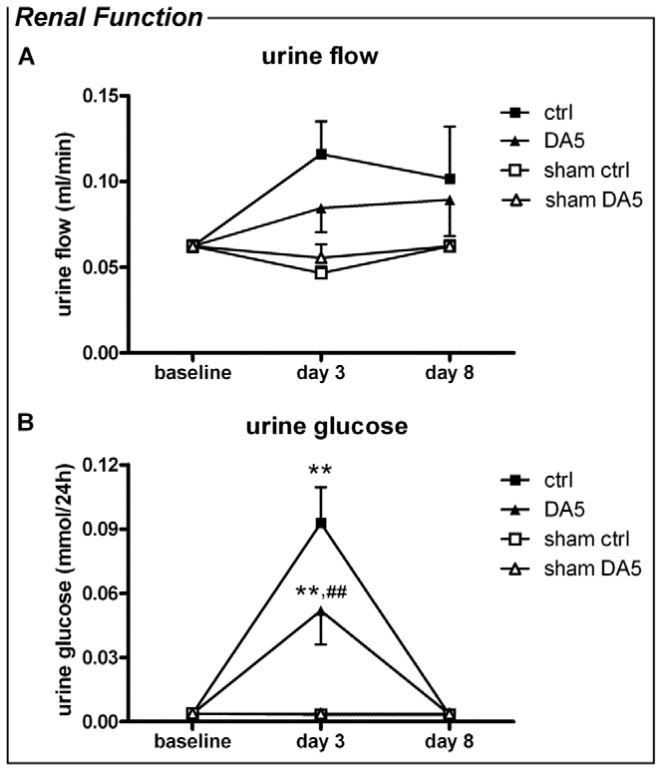
Diannexin (DA5) improves kidney function after renal IRI. Urine flow and urine glucose were measured at baseline, and 3 and 8 days after renal IRI. Twenty minutes of renal IRI significantly increased both parameters when compared to sham-operated mice on day 3. On day 8, recovery of renal function was observed. (A) When compared to vehicle-treated mice (ctrl), DA5-treatment reduced glucosuria on day 3. (B) Urine flow was increased on day 3 *post*-ischemia in vehicle-treated controls, which was normalized by DA5 treatment. Values are means ± SEM. *P<0.05, **P<0.01 *vs.* sham; ^#^ *P<*0.05, ^##^ *P<*0.001 *vs.* DA5 group. n = 5 for sham operated mice and n = 10 for all other groups.

On the functional level, renal damage in mice undergoing I/R was reflected by a significant increase in plasma urea (sham 10.8±1.7 *vs.* vehicle-treated 17.9±8.0 mmol/l), renal glucosuria and an increase in urine flow (Figure 4) on day 3 post-op, when compared to sham-operated mice. Plasma glucose did not differ between sham-operated mice (10.0±1.0 mM) and vehicle- or DA5-treated mice (8.9±1.9 and 11.4±5.1 mM, respectively). Recovery of renal function was observed on day 8, when plasma urea (sham 10.8±1.7 *vs.* vehicle-treated 13.7±5.8 mmol/l), urine glucose and urine flow (Figure 4) returned to baseline levels. DA5 treatment had beneficial effects on tubular function on day 3 after IRI: glucosuria was reduced when compared to vehicle-treated mice (*P<*0.001; Figure 4A). DA5 also prevented the increase in urine flow observed in vehicle-treated mice 3 days after I/R-induction (*P<*0.05; Figure 4B). Water intake after surgery was not different from baseline (3.8±0.7 ml) for either DA5 or vehicle treated animals (2.4±1.5 and 3.2±1.6 ml, respectively, *P*>0.05). No effect of DA5 on plasma urea was observed.

### Biodistribution and elimination of ANXA5 and DA5 after i.v. administration

We compared the biodistribution and elimination of DA5 with monomer ANXA5, in order to clarify the pharmacokinetic properties and imaging potential of DA5. Both proteins were labelled with Technetium-99m (^99m^Tc) via hydrazinonicotinamide (HYNIC) conjugation.

**Figure 5:**
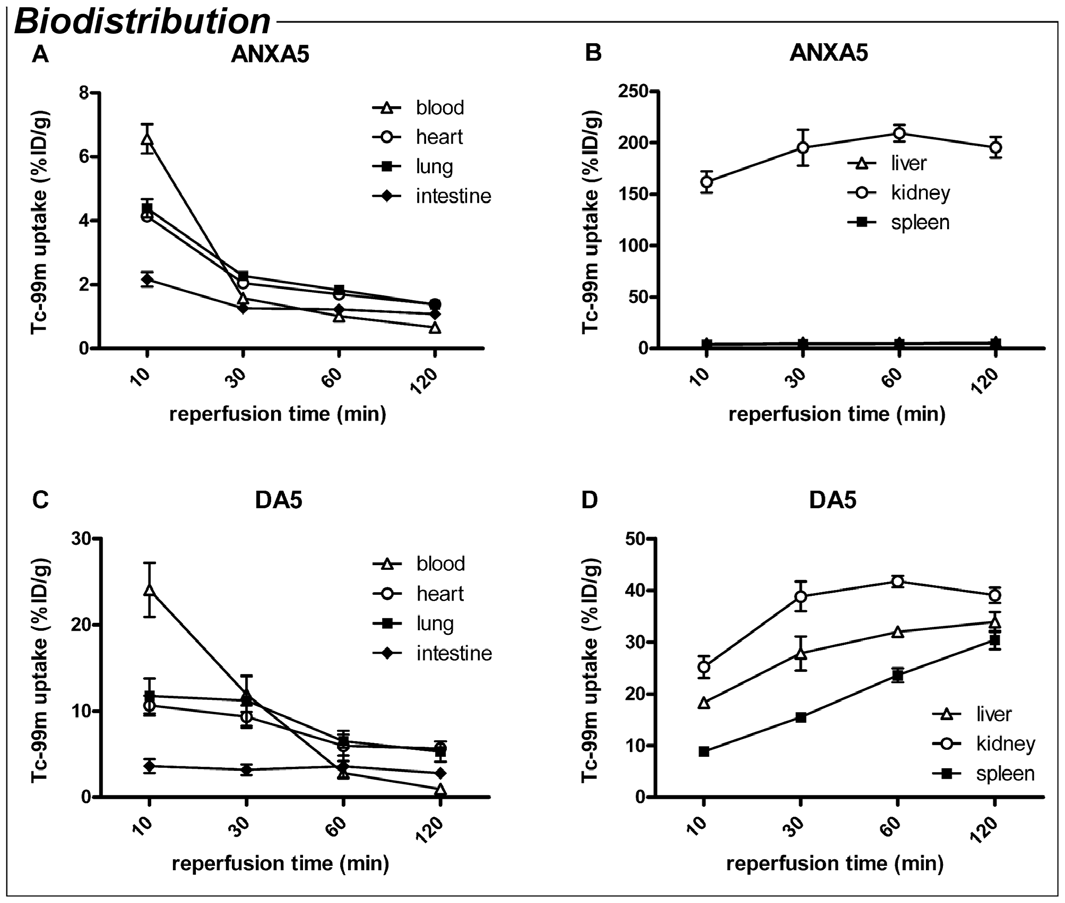
Biodistribution of Annexin A5 (ANXA5) and Diannexin (DA5) after i.v. injection, over a time course of 2 h. Uptake of ^99m^Tc in vital organs is presented as % of the injected dose (ID) per gram. (A) Plasma levels of ANXA5 declined steeply 10 to 30 min after injection. (B) Accumulation was most pronounced and rapid in the kidney, while low levels of ANXA5 were found in liver and spleen. (C) DA5 blood levels at t = 10 min were over 3 times higher than of ANXA5 and declined until 1 h after injection. (D) Accumulation of the probe was detected in kidney, liver and spleen. Values are means ± SEM. n = 3 mice per treatment.

Ten min after i.v. injection, blood levels of ^99m^Tc-ANXA5 were measured at 6.5% ± 0.8 of the injected dose per gram (%ID/g; Figure 5A). This level decreased rapidly over the next 20 min to 2%ID/g, and declined further during the remaining 90 min. In line with previous reports, we found that ^99m^Tc-ANXA5 accumulation was most pronounced and rapid in the kidney (Figure 5B and Figure S1A). ^99m^Tc-ANXA5 accumulation in liver and spleen was slow and did not exceed 6 %ID/g. Tracer concentration in intestine, lung and heart was below 2%ID/g after two h. For ^99m^Tc-DA5 (Figure 5C), blood levels after 10 min were >3 times higher than those of ^99m^Tc-ANXA5 (24.5±3.4%ID/g *vs.* 6.5±0.8%ID/g; *P<*0.01) and declined to just below 3%ID/g after 1 hour. Accumulation of ^99m^Tc-DA5 was prominent in kidney, liver and spleen (Figure 5D and Figure S1B), suggesting that ANXA5 and DA5 have different routes of elimination and/or metabolism. Uptake in heart and lung tissue was ∼10%ID/g after 10 min, but decreased in chorus with declining blood levels, suggesting that the ^99m^Tc-DA5 content of these tissues was dependent on the concentration in the blood.

### DA5 targets ischemic tissues after hind limb IRI

**Figure 6:**
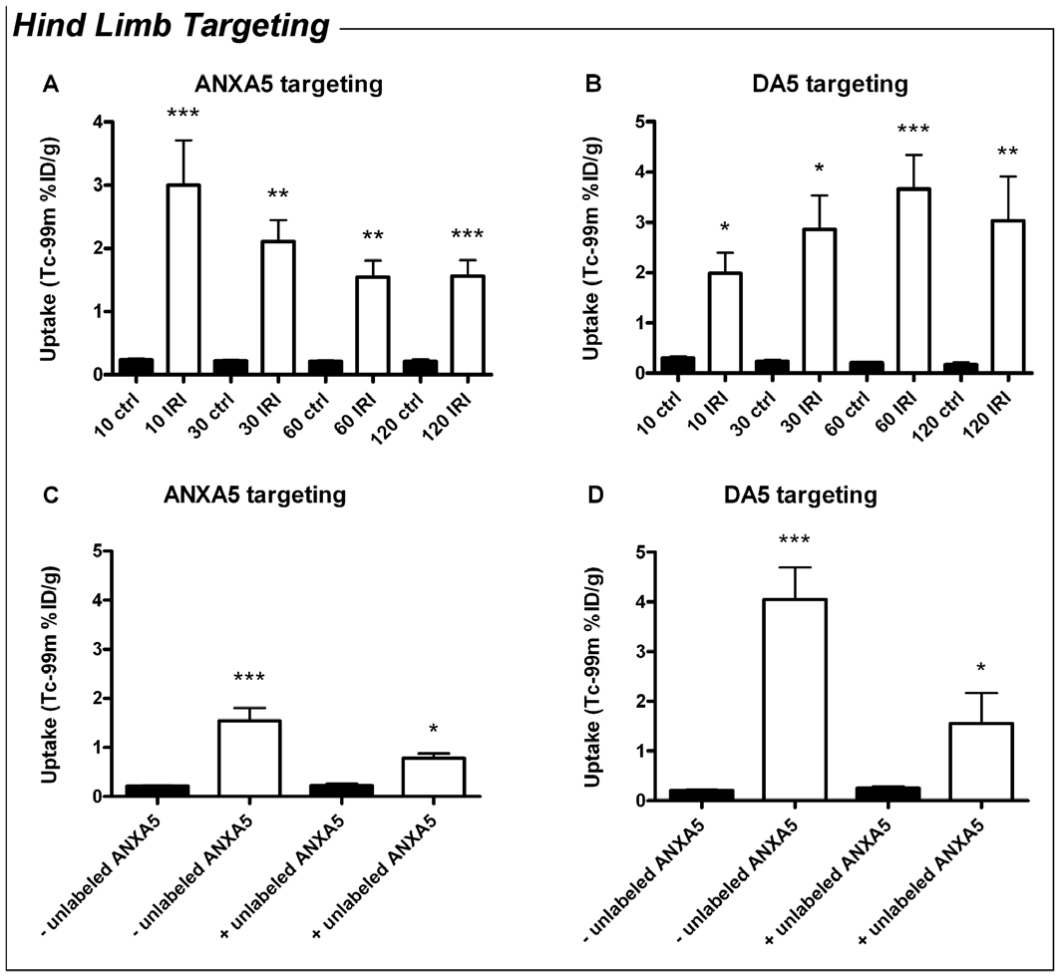
Diannexin and annexin A5 share the same target in the muscle after ischemic exercise. (A–B) Uptake of annexin A5 (ANXA5, panel A) and Diannexin (DA5, panel B, n = 4–8) in control limbs (filled bars) and limbs subjected to ischemic exercise and reperfusion (open bars). (A) When compared to the control limb, targeting of ^99m^Tc-ANXA5 was increased in hind limb muscle tissue subjected to ischemic exercise and 10, 30, 60 and 120 min of reperfusion. (B) Similarly, ^99m^Tc-DA5 was increased in hind limb muscle tissue subjected to ischemic exercise and 10, 30, 60 and 120 min of reperfusion. Values are means ± SEM. *P = 0.03 *vs.* ctrl group (paired t-test); **P<0.001, ***P<0.0001 *vs.* ctrl group (one-way ANOVA). n = 3–4 for 10, 30 and 120 min reperfusion and n = 7–8 for 60 min reperfusion. (C–D): effect of pre-treatment with unlabeled annexin A5 (ANXA5) on the targeting of labelled ANXA5 and Diannexin (DA5) to control muscle (filled bars) and ischemically exercised muscle (open bars). ^99m^Tc-ANXA5 (C) and ^99m^Tc-DA5 (D) target to the hind limb muscle after ischemic exercise and 60min of reperfusion. This targeting was blocked by administration of 450 µg of unlabeled ANXA5 prior to ischemia. In the presence of unlabeled ANXA5, targeting to the ischemic muscle was not significantly different from control muscle. Values are means ± SEM. **P<0.001 *vs.* control muscle; ^#^ *P<*0.05 *vs.* ischemic muscle - unlabeled ANXA5. n = 3 for ANXA5 pre-treated mice and n = 6–7 for control mice.

Since previously published data emphasize the high affinity of DA5 for exposed PS [21], we investigated whether DA5 targets to ischemic tissues and tested if DA5 and ANXA5 share similar binding sites, reflecting specific targeting to PS. Targeting of both ^99m^Tc-ANXA5 and ^99m^Tc-DA5 was investigated in an ischemic exercise model of the mouse hind limb, mimicking our experimental set up used to detect ANXA5 targeting after ischemic exercise in humans *in vivo* [10]. We injected the radiolabeled annexin analogues immediately prior to reperfusion and measured the targeting of ^99m^Tc-ANXA5 and ^99m^Tc-DA5 during 2 h of reperfusion. Muscle tissue from the untreated contralateral limb served as negative control. ^99m^Tc-ANXA5 targeting to the muscle subjected to I/R was significantly elevated at all measured time points (Figure 6A). This targeting appeared to decrease during the first 30 min of reperfusion, which may be due to reactive hyperaemia and decreasing blood levels of ^99m^Tc-ANXA5 (Figure S1A). However, targeting reached a steady-state between 30 and 120 min, indicating high affinity binding of ^99m^Tc-ANXA5 in muscle tissue after I/R. For ^99m^Tc-DA5, targeting to the I/R muscle increased over time, reaching a maximum after 60 min of reperfusion (Figure 6B). Thus, ^99m^Tc-DA5 targeting appeared to accumulate over time, even though ^99m^Tc-DA5 blood levels decreased. Furthermore, targeting of ^99m^Tc-DA5 was significantly higher than that of ^99m^Tc-ANXA5 after 1 h (1.5±0.3%ID/g *vs.* 3.7±0.7%ID/g; *P<*0.05) and 2 h (1.6±0.3%ID/g *vs.* 2.9±0.6%ID/g; *P<*0.05).

In a second set of experiments, we showed that targeting of both ^99m^Tc-ANXA5 and ^99m^Tc-DA5 to the ischemic muscle was blocked by administering 450 µg of unlabelled monomer ANXA5 prior to ischemia and the injection of labelled substrate (Figure 6C, D). This finding confirms our hypothesis that ANXA5 and DA5 have the same binding sites, reflecting specific targeting to PS.

## Discussion

The present study is the first to demonstrate that the ANXA5 homodimer DA5 decreases renal damage after IRI, in terms of kidney morphology, inflammation, gene expression and renal proximal tubule function. Furthermore, we show that DA5 targets specifically to exposed PS in ischemic tissue *in vivo.*

The therapeutic effects of DA5 have been studied previously in rodent models of hepatic and pancreatic IRI and transplantation [24]–[26], and in an IRI model of rat cremaster muscle [27]. However, favourable effects on renal IRI were not yet demonstrated, even though its vulnerability to I/R and its impact on prognosis make the kidney an ideal candidate for preventive treatment against IRI. Clinical conditions that jeopardize renal perfusion are frequently encountered and can often be predicted, *e.g.* in elective major surgery and kidney transplantation. In such cases, an intervention that should preferably be timed at the moment of I/R is practical. Here, we used a murine model of renal IRI to show that a single i.v. administration of DA5 (200 µg/kg b.w.) 15 min before renal IRI decreases proximal tubule damage, nearly abolishes leukocyte influx, decreases transcription and expression of renal injury markers and ameliorates renal function. These effects were observed after 3, and up to 8 days of reperfusion, indicating that the beneficial effect of DA5 outlasts the acute phase of I/R and lasts for several days.

We observed beneficial effects of DA5 on renal morphology, leukocyte influx, as well as functional changes in urine flow and urine glucose. There is increasing evidence that PS externalisation on the endothelial cell membrane is an early event in IRI, giving rise to the adhesion and aggregation of activated leukocytes and platelets in the microcirculation. This in turn exacerbates injury due to *e.g.* ROS formation, the release of proteolytic enzymes from adherent or transmigrated leukocytes, and diminished microcirculatory perfusion. Our findings show that DA5 is able to shield exposed PS, and that leukocyte influx into the ischemic kidney is reduced in DA5-treated mice. Similar to monomeric ANXA5, the anti-inflammatory and anti-thrombotic properties of DA5 are thought to originate from its ability to shield PS, thereby reducing leukocyte adherence and transmigration, as well as preventing the formation of the pro-thrombinase complex. Prevention of leukocyte and platelet adhesion in turn leads to improved microcirculation, while reduced leukocyte infiltration diminishes the secondary inflammatory response. The precise mechanism of action is currently unknown; similar to ANXA5, DA5 may alter the accessibility and/or membrane expression of adhesion molecules through steric hindrance, and/or influence endocytosis and phagocytosis. For ANXA5, these effects are contributed to PS-binding and subsequent 2D crystallisation of the protein on PS-exposing membranes. It is at present unknown whether DA5 exhibits 2D-crystallization. Lastly, we cannot exclude that the anti-inflammatory effects of DA5 could be receptor-mediated, or based on intracellular actions of the protein. As was recently described for annexin A1, receptor activation on the epithelial cells and/or leukocytes can greatly reduce leukocyte adhesion and infiltration [28]. Such a receptor for ANXA5 is putative at best, although ANXA5 has been shown to be internalised by living cells [29] and influence PKC activity. For DA5 it is at present unknown whether these processes occur and therefore, full understanding of the mechanism requires further investigation.

Apart from the morphological and functional outcome measures mentioned above, we also assessed the renal damage markers KIM-1 and NGAL in our mouse model. The great advantage of these markers is that they are early, sensitive, diagnostic indicators of kidney injury when compared to conventional biomarkers, *e.g.* plasma creatinine and blood urea nitrogen [23], [22]. Moreover, in mice these markers are preferred over creatinine clearance (CCr), since the impact of tubular creatinine excretion on CCr is even larger in mice than in humans, raising questions regarding the reliability of creatinine for measuring renal function in these animals [30]. Furthermore, these markers support our measurements of urine flow and urine glucose, which are important, although not standard, indicators of (proximal) tubule damage, since glucosuria indicates proximal tubular dysfunction, but does not directly reflect a change in GFR. We show here that KIM-1 and NGAL are expressed at low levels in healthy renal tissue, but increase dramatically in the *post-*ischemic kidney. DA5 treatment reduced the expression and transcription of KIM-1 after 3 and 8 days of reperfusion. It has been shown *in vivo* that the ectodomain of KIM-1 is shed into the urine in rodents and humans after proximal tubular kidney injury. This provides us with an important tool for future research to translate current findings to humans *in vivo*. We found no significant increase in NGAL mRNA expression, but this marker has been reported to reach its maximum transcription at an earlier time-point [22]. Decreased levels of KIM-1 and NGAL mRNA were also observed in a pilot study of unilateral renal IRI, in which DA5 was administered directly upon reperfusion, rather than 15 minutes before ischemia (unpublished observations; Wever et al. 2009). Interestingly, KIM-1 has been described as a PS receptor involved in the conversion of epithelial cells to semi-professional phagocytes [31]. Thus, KIM-1 may actually play a mechanistic role in kidney injury downstream of the initial PS exposure. Shielding of PS by DA5 could therefore lead to a decrease in KIM-1-mediated phagocyte recruitment, thereby reducing damage.

In addition to our therapeutic findings, this study is the first to show comparative data on the biodistribution and elimination of ANXA5 and DA5. These data are of vital importance for future clinical studies, since the pharmacokinetic and imaging properties of DA5 are largely dependent on these parameters. In cardiovascular medicine and oncology, detection of apoptosis, which is accompanied by PS exposure, may provide important diagnostic and therapeutic information. Previous reports on the biodistribution and clearance of ^99m^Tc-HYNIC-ANXA5 in humans and rodents showed rapid accumulation of ANXA5 in kidney (∼50% of the injected dose; ID) and liver (∼12%ID) after 1–3 h. Furthermore, kinetic data for ANXA5 in the human circulation indicate a short plasma half-life of 24 min, while clearance occurred almost exclusively via urinary excretion [32], [33]. Clearance of DA5 has been studied previously in rats using a ^125^I-DA5-conjugate. DA5 clearance fitted a two-compartment model, revealing a 1^st^ and 2^nd^ phase half life of ∼12 min and ∼6.5 h, respectively [24]. In the present study, we evaluated the ^99m^Tc-DA5 pharmacokinetics together with ^99m^Tc-ANXA5, and confirmed the longer half-life of ^99m^Tc-DA5 as compared to ^99m^Tc-ANXA5. For imaging purposes, generally a short half-life is preferred, because this allows rapid clearance of the tracer from the background. However, the circulatory half-life is also the driving force for accumulation of the tracer in the target tissue. The circulatory half-life of Annexin A5 is considered to be too short for many imaging purposes, as discussed in e.g. [34], [35]. The slightly longer half-life of DA5, combined with its higher affinity for PS, makes the dimer more suited to image PS exposure in tissues *in vivo* than monomer ANXA5. We found that ^99m^Tc-DA5 shows physiologic uptake in liver, spleen and kidney. With respect to the imaging properties of DA5, this nonspecific uptake in abdominal organs may hamper visualization of apoptosis in tissues in the vicinity of these organs, although in most cases single photon emission computed tomography (SPECT) imaging will allow distinction between physiologic uptake and uptake in tissues nearby. Biodistribution studies using various DA5 conjugate/radionuclide combinations are required to optimize the imaging potential of DA5 [36].

In our experiments on DA5 and ANXA5 targeting, we found that ^99m^Tc-DA5 accumulates in the hind limb muscle during the first 2 h after ischemic exercise. Targeting of DA5 increased over time and was significantly higher than that of ANXA5, which translates previous *in vitro* observations stating that DA5 has a higher affinity for PS than ANXA5 [21], to the *in vivo* setting. Furthermore, we showed that ^99m^Tc-DA5 targeting to ischemic tissues is PS-specific, since its binding was inhibited by unlabeled ANXA5. This observation indicates that DA5 and ANXA5 share the same binding sites in the ischemically exercised muscle and it is well appreciated that ANXA5 specifically binds to PS [37], [16].

The importance of exposed PS in ischemic injury is grounded by our evidence that PS-targeting by DA5 attenuates IRI in the kidney. The therapeutic effects of DA5, such as those shown in our renal IRI model, are thought to be local rather than systemic and may be enhanced by DA5 enrichment in ischemic tissues. The higher *in vivo* affinity of DA5 for PS likely results in a higher efficacy of this drug to prevent IRI as compared to ANXA5. The therapeutic dose and maximal tolerable dose of DA5 in humans have not yet been determined, but DA5 administration in patients in single doses up to 400 µg/kg was reported to be without serious complications [27]. Hemorrhagic complications in particular could, theoretically, be of concern. Of note, DA5 administration did not induce any notable *post-*operative bleeding complications in any of our mice (observations in >60 mice, data not shown). Overall, effects of ANXA5 and DA5 on systemic haemostasis have been reported to be very minimal [21], [19], [24]. It is likely that reduced inflammation, by diminished leukocyte adhesion, and consequently reduced tissue damage are involved in protection by DA5. This makes DA5 a promising new therapeutic agent to prevent IRI in a wide range of clinical settings where renal perfusion is at risk. Furthermore, DA5 may offer new possibilities in the field of PS-imaging in e.g. atherosclerosis, and used to provide valuable insights in the pathogenesis of the disease. As a first step towards clinical studies on DA5, the homodimer has recently completed a Phase II clinical trial in kidney transplantation [26].

## Materials and Methods

### Chemicals

DA5 was an unrestricted gift from Alavita Pharmaceuticals Inc. (Mountain View, CA). ANXA5 was obtained from Theseus Imaging (Boston, MA). SYBR Green® and Taqman® Universal PCR Master Mix and NGAL and β-actin primer probe sets (Mm01324470_a1 and 4352933E) were from Applied Biosystems, Zwijndrecht, The Netherlands.

### Animals

All procedures involving animals were approved by the Committee for Animal Experiments of the Radboud University Nijmegen Medical Centre (experiment ID's DEC2008121 and DEC2009006). Male FVB (Friend leukaemia virus B strain) mice were obtained from Charles River Germany at 5–7 weeks of age, weighing 18–22 grams on arrival. Animals were housed under standard specific pathogen free housing conditions at the Central Animal Facility Nijmegen. Up to 10 mice per cage were housed in Macrolon cages (Techniplast, Buguggiatta, Italy), supplied with woody bedding, shelters and treadmills. Mice had *ad libitum* access to drinking water and standard chow (RMH-TM, Hope Farms, Woerden, The Netherlands). The environmental temperature was regulated at 22°C, with a relative humidity of ± 45% and a 12/12 h day/night cycle in artificial lighting with white lights on at 06:00 h. Radio sound was played during the day period. Air was refreshed at the rate of approximately six times per hour. Mice were allowed to acclimatize for at least one week before surgery.

### Metabolic cages

Single mouse metabolic cages (Techniplast, Buguggiate, Italy) were used to assess renal function 7 days pre-op and 2 or 7 days post-op. Prior to 24 h housing in metabolic cages, mice were weighted and 0.5 ml physiological salt solution was administered s.c. to prevent dehydration. To prevent hypothermia, room temperature was raised to 24°C with a relative humidity of 53–68%. After 24 h, body weights (b.w.) were recorded and a blood sample was collected via the retro-orbital sinus under isoflurane anaesthesia (5% in O2/N2O).

### Surgical procedures

All experiments were performed between 08.00 and 16.00 h. Surgical procedures were conducted using standard aseptic surgical techniques and all microsurgical instruments were sterilized using a dry bead sterilizer (Inothech, Dottikon, Switzerland). Animals were placed on a sterile drape overlying a heating pad to maintain body temperature at 37°C, which was monitored continuously using a rectal thermometer probe. Body weights were recorded prior to surgery. Anaesthesia was induced with 5% isoflurane in O2/N2O and maintained at 2.5–3%. Depth of anaesthesia was assessed by toe and tail pinch.

### DA5 and ANXA5 biodistribution and elimination

^99m^Tc-DA5 and ^99m^Tc-ANXA5 were prepared as described previously [10] at a specific activity of 5–25 µCi/µg. The radiolabelled products were purified by PD-10 column. The radiochemical purity was found to be >90%, as determined by instant thinlayer chromatography. PS-binding of the ^99m^Tc-DA5 and ^99m^Tc-ANXA5 conjugates was confirmed using denaturated erythrocytes *in vitro* as described previously [38].

Mice were anaesthetised and 200 µg/kg b.w. of ^99m^Tc-DA5 or ^99m^Tc-ANXA5 was administered i.v. via the tail vein. For scintigraphic imaging of whole body ^99m^Tc biodistribution at 0, 10, 30, 60 and 120 min, mice (n = 3 per treatment) were placed on the low-energy collimator of a single-headed gamma camera (Orbitor, Siemens, Hoffman Estates IL). Animals were re-anaesthetized for measurements at 30, 60 and 120 min, after which they were euthanized through exsanguination. The ^99m^Tc content of blood and various tissues was determined by counting tissue samples in a well-type gamma counter (Wizard, Pharmacia-LKB).

### Hind limb ischemic exercise model

Anaesthesia was induced and mice (n = 3–4 for 10, 30 and 120 min reperfusion and n = 7–8 for 60 min reperfusion) remained anesthetized until sacrifice. The *Nervus ischiadicus* of the right hind limb was exposed via an incision and blunt dissection through the right thigh. A 1 mm silicone copper wire, with a stripped section of 2–3 mm at 2 cm from its tip, served as positive electrode. The stripped section was placed underneath the nerve and the wire was tunnelled s.c. to exit at the tail base. A second length of wire with stripped ends was used as negative electrode, inserted through the heel of the right hind limb. Both electrodes were connected to the pulse generator, after which 1 test pulse was applied manually.

Approximately 20 min after the induction of anaesthesia, hind limb ischemia was induced by placing an orthodontic rubber band (ORB) around the proximal thigh using a McGivney ligator applicator. Dark pink discoloration of the ischemic toes and absence of swelling indicated successful occlusion of arterial and venous blood flow. After ORB application, the *N. ischiadicus* was stimulated electrically with 20 pulses at 10 sec intervals. The minimal electric current needed for a maximal contraction and the number of pulses needed to reach exhaustion of the muscle were determined in a pilot study. Stimulus parameters were controlled by an S48 stimulator, connected to a SIU5 telefactor stimulus isolation unit and a constant current unit (all by Grass, Astro-Med, Rodgau, Germany). Pulses were generated at 0.1 tps train rate, 250 ms train duration, 150 pps stimulus rate, 0.1 ms stimulus delay and 0.1 ms duration at 150 volt. The constant current unit was used to adjust the electric current output to 0.9 ampere.

After completion of the stimulation protocol, ^99m^Tc-DA5 or ^99m^Tc-ANXA5 was administered i.v. via the tail vein (200 µg/kg b.w.; 20 µCi per mouse). Immediately after DA5 or ANXA5 administration, blood circulation was restored by cutting the ORB (total ischemia time was 5 min). After the desired reperfusion time (10, 30, 60 or 120 min), mice were sacrificed by cervical dislocation, and the ^99m^Tc concentration of the hind limb muscles was determined in a gamma counter (Wizard, Pharmacia-LKB).

The effect of a preceding injection of unlabeled ANXA5 (450 µg per mouse) was tested in two separate groups of mice (n = 3 per treatment). The unlabeled ANXA5 was injected i.v. via the tail vein immediately prior to hind limb occlusion. The rest of the protocol was as described above.

### Renal I/R model

For recovery experiments, ethical guidelines prescribe the use of analgesia with either non-steroidal anti-inflammatory drugs or opiates. Since opiates are known to influence IRI signalling, carprofen (5 mg/kg b.w.) was selected as analgesic in all experimental and control groups, even though this drug may possibly have effects on renal function and inflammatory cells. Carprofen was administered s.c. 30 min prior to surgery. DA5 (200 µg/kg b.w.) or vehicle (physiological salt solution) was administered i.v. via the tail vein [25], [24]. Hereafter, anaesthesia was induced, the incision site was shaved, iodized and covered with sterile surgery foil, after which mice were laparotomized. The jejunum, ileum and cecum were temporarily placed outside the abdomen, wrapped in gauze and kept moist with physiological salt solution. For both kidneys, the renal vein and artery were isolated and clamped for 20 min using non-traumatic microvascular clamps (S&T, Neuhausen, Switzerland). Time between DA5 or vehicle administration and clamping was ±15 min. Complete occlusion of blood flow and reperfusion after clamp release were confirmed visually by swift dis- and re-coloration of the kidney. After clamp removal, the intestine was placed back, the abdominal wall and skin were sutured and the animals were allowed to recover in a clean housing cage. Analgesic (carprofen, 5 mg/kg b.w.) was administered s.c. 24 h and 48 h post-op. On day 3 or day 8 post-op, mice were anesthetized with 5% isoflurane in O2/N2O and sacrificed through exsanguination (8–10 mice per day per treatment). Sham operated mice (n = 5 per treatment) underwent all procedures described above, except for clamp placement, and were sacrificed on day 3 post-op.

### Tissue handling and renal histology

Blood samples were collected in EDTA tubes and centrifuged for 15 min at 3000 g to obtain plasma. Plasma and urine aliquots were snap frozen in liquid nitrogen and stored at −80°C until further use. For immunohistochemistry, tissue was embedded in Tissue-Tek Optimal Cutting Temperature (OCT) compound (Sakura Finetek, Zoeterwoude, The Netherlands) and snap frozen in liquid nitrogen. Tissues were stored at −80°C until further use. For histological studies, tissue was fixed in 4% paraformaldehyde for at least 24 h.

For light microscopy of the renal cortex, ½ kidneys were dehydrated and embedded in paraffin. For damage scoring, sections of 5 µm were stained with periodic acid-Schiff (PAS). For each kidney, 4 sections taken at different latitudes were scored for damage of the renal cortex and averaged. Damage scoring was performed on a scale from 0 to 5, with 0 signifying no proximal tubule damaged, and 5 indicating that all tubules were damaged (see also Figure 1A-D and legend). For leukocyte counting, 5 µm sections were stained with Leder's esterase, which stains all myeloid granulocytes and mast cells. Images of 10 random fields of the renal cortex were obtained at 400x magnification for each kidney. Granulocytes were counted in these 10 fields and averaged for each kidney. For both damage and granulocyte scoring, the investigator was blinded for the experimental group to which the mice had been assigned.

### Membrane preparation and Western blotting

Total membrane fractions were obtained using a micro-dismembrator (Sartorius BBI Systems GmbH, Melsungen, Germany), as described previously [39]. Frozen kidneys were pulverized (2000 rpm, 30 s) and transferred to ice-cold TS buffer (10 mM Tris-HCl, 250 mM sucrose) including protease inhibitors (complete Mini, Roche, Mannhein, Germany). From these suspensions, a supernatant containing the total protein fraction was prepared through centrifugation (30 min×12000 g, 4°C). Protein concentrations were determined with a standard protein assay (Biorad, Veenendaal, The Netherlands).

For Western blot analysis, samples were blotted in a blinded fashion to avoid bias and were therefore randomly distributed over several blots. Ten µl samples corrected for protein amount were solubilized in Laemmli sample buffer, heated at 95°C for 5 min, separated on a 10% polyacrylamide gel, and transferred to a nitrocellulose membrane using the Iblot system (Invitrogen, Breda, The Netherlands). Subsequently, the blot was blocked for 60 min with 5% nonfat dry milk powder in PBS supplemented with 0.1% Tween 20 (PBS-T), after which the blot was washed three times in PBS-T. The membrane was incubated overnight at 4°C with the primary antibodies: RbαH polyclonal KIM-1 (AbD serotec, Oxford, UK) diluted 1∶1000, RαM monoclonal NGAL (Santa Cruz, Heidelberg, Germany) diluted 1∶1000 and MαM β-actin (Sigma, Zwijndrecht, The Netherlands) diluted 1∶100.000. The blot was washed twice for 20 min with PBS-T, rinsed three times with PBS and then blocked for 30 min as described above. The blot was then washed three times for 20 min with PBS-T and incubated at room temperature for 60 min with secondary antibodies alexa680-GαRb, alexa680-GαR (both Invitrogen, Breda, The Netherlands) or alexa800-GαM (Rockland, Gilbertsville, PA), diluted 1∶5000. Finally, the blot was washed twice for 20 min with PBS-T and rinsed twice in PBS. Proteins were visualized using the Odyssey Infrared Imaging Scanner (LI-COR®, Lincoln, NE). Semi-quantitative analyses were performed by correcting relative fluorescence from the proteins of interest for housekeeping protein (ß-actin) fluorescence.

### RNA isolation and cDNA reaction

RNA isolation and cDNA reaction were performed on ½ kidney from each mouse as described previously [39]. RTQ-PCR was performed in duplicate on approximately 1 ng cDNA, using the ABI/PRISM 7900HT Gene Expression Micro Fluidic Card (Applied Biosystems, Foster City, CA) according to the manufacturer's instructions. Primers for KIM-1 (forward 5′- GCTATGCTCTCCCTCACGCCA-3′ and reverse 5′- CTCTTTGATGTCACGCACGAT-3′) and β-actin (forward 5′-CCTCCACTCCTCCAACATCTACA-3′ and reverse 5′-ACTGTCCTTAGGGTAGGGT-3′) were developed using the primer express software (Applied Biosystems, Zwijndrecht, The Netherlands). cDNA amplification of KIM-1 and β-actin was performed in 2x SYBR Green® PCR Master Mix, supplemented with 1.5 µl of 10 µM forward and reverse primer in a total reaction volume of 25 µl. cDNA amplification of NGAL and β-actin was performed in Taqman® Universal PCR Master Mix, supplemented with 20x solution of each primer probe set.

The thermal cycling conditions were 2 min at 50°C and 10 min at 95°C, followed by 40 cycles of 10 s at 95°C and 1 min at 60°C. PCR reactions were analyzed using 700 System Sequence Detection Software (version 1.2.3, Applied Biosystems).

### Data analysis

Data are given as means ± SEM. Number of animals used is given in the figure legends. Mean values were considered to be significant when P<0.05 by use of a two-way ANOVA, one-way ANOVA or Student's t-test where applicable. Software used for statistical analysis was Graphpad Prism® (version 5.02 for Windows; Graphpad Software, San Diego, CA).

## Supporting Information

Figure S1 **Whole body scintigraphy and blood elimination of i.v. 99mTc-Annexin A5 (ANXA5; top panel) or 99mTc-Diannexin (DA5; bottom panel), over a time course of 2 h.** (A) Accumulation of ANXA5 in kidney and loss of signal from the peripheral circulation occur rapidly after injection. Excretion of ANXA5 via the urine is evident 30 to 120 min after injection (arrows indicate bladder). (B) Accumulation of DA5 is detected in kidney, liver and spleen immediately after injection. Note the prolonged activity in the peripheral circulation, indicating the prolonged half-life of DA5. (C, D) Elimination curves of ANXA5 (C) and DA5 (D) as calculated from blood 99mTc activity, 10 to 120 min post-injection. n = 3 mice per treatment.
(PDF)
