# Supplementary material for: Diannexin Protects against Renal Ischemia Reperfusion Injury and Targets Phosphatidylserines in Ischemic Tissue
Source: PLoS One. 2011 Aug 30;6(8):e24276. doi: 10.1371/journal.pone.0024276 (PMC3168880; doi:10.1371/journal.pone.0024276)
Supplement: Figure S1 — Whole body scintigraphy and blood elimination of i.v. 99mTc-Annexin A5 (ANXA5; top panel) or 99mTc-Diannexin (DA5; bottom panel), over a time course of 2 h. (A) Accumulation of ANXA5 in kidney and loss of signal from the peripheral circulation occur rapidly after injection. Excretion of ANXA5 via the urine is evident 30 to 120 min after injection (arrows indicate bladder). (B) Accumulation of DA5 is detected in kidney, liver and spleen immediately after injection. Note the prolonged activity in the peripheral circulation, indicating the prolonged half-life of DA5. (C, D) Elimination curves of ANXA5 (C) and DA5 (D) as calculated from blood 99mTc activity, 10 to 120 min post-injection. n = 3 mice per treatment. (PDF) [file pone.0024276.s001.pdf]

ONLINE SUPPLEMENT

Supplemental Figure 1

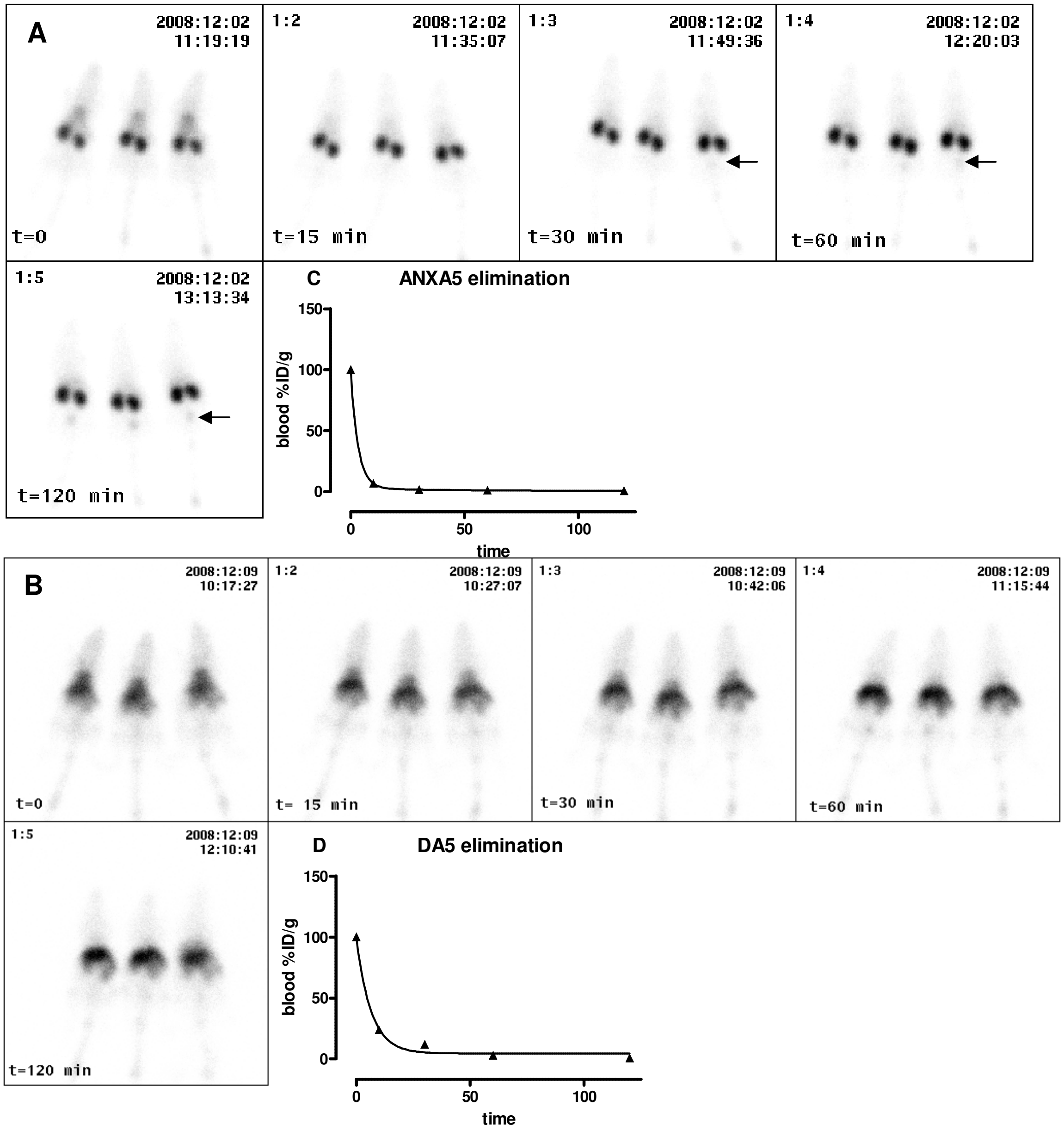

Whole body scintigraphy and blood elimination of i.v. ^{99m}Tc-Annexin A5 (ANXA5; top panel) or ^{99m}Tc-Diannexin (DA5; bottom panel), over a time course of 2 h. (**A**) Accumulation of ANXA5 in kidney and loss of signal from the peripheral circulation occur rapidly after injection. Excretion of ANXA5 via the urine is evident 30 to 120 min after injection (arrows indicate bladder). (**B**) Accumulation of DA5 is detected in kidney, liver and spleen immediately after injection. Note the prolonged activity in the peripheral circulation, indicating the prolonged half-life of DA5. (**C, D**) Elimination curves of ANXA5 (**C**) and DA5 (**D**) as calculated from blood ^{99m}Tc activity, 10 to 120 min *post*-injection. n=3 mice per treatment.
